# Examining the associations of self-control with physical fitness, cardiometabolic health, and well-being in young people aged 9–13 years

**DOI:** 10.3389/fcogn.2026.1755134

**Published:** 2026-04-01

**Authors:** Anna Dunn, Ryan A. Williams, Karah J. Dring, Simon B. Cooper, Ruth Boat

**Affiliations:** Sport Science Department, Sport, Health and Performance Enhancement (SHAPE) Research Centre, School of Science and Technology, Nottingham Trent University, Nottingham, United Kingdom

**Keywords:** cardiometabolic health, physical fitness, self-control, well-being, young people

## Abstract

**Background:**

Previous research has demonstrated that high self-control is associated with cardiorespiratory fitness and physical activity in young people. However, the associations of self-control with objective health measures (blood pressure, blood glucose, and plasma insulin), different components of physical fitness (motor and musculoskeletal fitness), motor competence and well-being remain largely unexplored.

**Methods:**

Utilizing a cross-sectional design, 149 young people (aged 9–13 years) from the East Midlands, England completed measures of self-control, physical fitness, adiposity, well-being, motor competence, and cardiometabolic health. Univariate Pearson correlations, best-subset regression and simple linear regression analyses were conducted.

**Results:**

High self-control was associated with better well-being (*r* = 0.43, *p* < 0.001) and higher levels of cardiorespiratory fitness (*r* = 0.21, *p* = 0.012), musculoskeletal fitness (*r* = 0.19, *p* = 0.020), and motor fitness (*r* = 0.23, *p* = 0.004). Best subset regression modeling identified the combination of predictors that best explained the variance in self-control, which included well-being, physical fitness (time on 4 x 10 m shuttle run), and HOMA-IR, with these effects independent of each other. Each individual component of well-being (physical well-being; *p* < 0.001, psychological well-being; *p* < 0.001, autonomy and parents; *p* < 0.001, and school environment; *p* < 0.001) was positively associated with self-control.

**Conclusions:**

The present study's findings support the notion that self-control could be a significant and attractive target for future interventions focused at encouraging healthy behaviors in young people, and ultimately enhancing well being.

## 1 Introduction

In spite of the well-recorded benefits of physical activity for young people, there has been no improvement in young people's physical activity levels (World Health Organization, 2024). In England alone, 50.9% of 5–16 year olds did not meet the recommended guidelines of at least 60 min of moderate to vigorous physical activity per day, in the 2024/2025 academic year (Sport England, 2025). These concerning statistics contribute to the increased prevalence of overweight and obesity (Bentham et al., 2017), with 41.7% of 10–11 year olds in England reported as overweight or obese (NHS England, 2023), which will contribute to an increased presence of risk factors for cardiovascular diseases and diabetes (Csige et al., 2018). Considering the low levels of physical activity and high rates of obesity among young people in England, it is imperative to investigate potential determinants of unhealthy behaviors (e.g., sedentary behavior and poor dietary habits), ultimately to support the development and design of future interventions focused on at improving the health and well-being of young people.

### 1.1 Self-control

Behavior theories have been developed to explain the factors (social, cognitive, emotional, economic) which influence why individuals engage in healthy or unhealthy behaviors (Davis et al., 2015). One such potential, yet relatively unexplored, determinant of healthy behavior choices is self-control. Self-control is the ability to consciously override dominant responses and resist temptations to help achieve long-term goals (Baumeister et al., 2007). Trait self-control is an individuals innate ability to exert self-control and it varies between individuals (Schmeichel and Zell, 2007), or it can differ for individuals across different situations (state self-control; Tangney et al., 2018). High trait self-control is associated with better regulation of emotions, improved psychological well-being, and enhanced interpersonal relationships (De Ridder et al., 2012).

### 1.2 Self-control and health-related behaviors

Given the above definition of self-control, it is unsurprising that trait self-control has been linked to health behaviors (Kinnunen et al., 2012). For instance, individuals with high self-control are more likely to adhere to diets (Keller and Hartmann, 2016) and have lower sedentary time (Wills et al., 2007), compared to individuals with low self-control. In young people, self-control has been shown to be associated with self-reported healthy behavior choices (e.g., increased physical activity, increased fruit and vegetable intake, and lower sedentary time; Junger and van Kampen, 2010; Wills et al., 2007).

Previous research has found a positive association between self-control and physical activity (Boat et al., 2022) and physical fitness (Boat et al., 2022; Brandon and Loftin, 1991; Schmidt et al., 2016), and a negative association between self-control and adiposity (Boat et al., 2022). For example, the findings from Boat et al.'s (2022) demonstrated that participants with high self-control, had higher physical fitness (determined by distance covered on multi-stage fitness test), spent more time in vigorous intensity physical activity, and had lower adiposity, compared to those who reported low self-control. However, it must be noted that cardiorespiratory fitness was the only component of fitness considered within these previous studies. A more holistic overview of fitness, considering other components of fitness (e.g., musculoskeletal and motor fitness) and how they relate to self-control has not yet been examined in young people. In addition, only one study in adults has examined the associations of self-control with multiple components of physical fitness (muscular and aerobic; De Ridder et al., 2012). Findings demonstrated that those with higher self-control performed better on an aerobic fitness test (12-min Cooper run) and musculoskeletal fitness tests (e.g., push-ups), compared to those with lower self-control. The study however, only recruited male adults; therefore, the associations between self-control and multiple components of physical fitness in young people remain unknown.

Similar to physical fitness, motor competence is important for understanding young people's physical activity patterns. There is currently limited evidence demonstrating the associations between individuals' motor competence and self-control. Motor competence is recognized as an important corelate of active lifestyles in young people (Lubans et al., 2010). Importantly, self-control has been associated with practice and persistence on a task (Muraven, 2010), which could be relevant to activities that involve repeated practice of motor skills (Papaioannou, 1997). (Pesce et al. 2016) found that following an exercise training program, gross motor skills were associated with greater inhibitory control in children. However, this study only assessed the ball-skills sub-component of gross motor skills, thus further research in the area is warranted to explore this association further.

It is also important to consider whether the potential aforementioned associations between self-control and physical activity, physical fitness, and motor competence translate to positive associations between self-control and health and well-being indicators. Heart rate variability (HRV; Maier and Hare, 2017) and cortisol concentrations (Shoal et al., 2003) have primarily been the health markers examined for their associations with self-control. Findings demonstrated that high self-control was associated with high HRV and low resting heart rate (Maier and Hare, 2017), with low self-control associated with low cortisol in preadolescents (Shoal et al., 2003). High HRV, low resting heart rate, and low cortisol are associated with good health, further demonstrating that high self-control is associated with beneficial health characteristics. Furthermore, (Miller et al. 2011) found that adolescents who reported having low self-control were more likely to exhibit risk factors for poor cardiometabolic health (e.g., high cholesterol and high blood pressure), compared with those who reported high self-control. However, it must be noted that a self-report questionnaire for cardiometabolic health was used. Thus, the findings from previous research suggest that high self-control may be associated with more favorable health outcomes. However, there is a scarcity of literature focusing on self-control and risk factors implicated in the etiology of cardiometabolic disease (e.g., blood pressure and insulin sensitivity). This is of importance given that risk factors for these key cardiometabolic conditions begin in childhood (Theodore et al., 2015); and thus intervention in young people is warranted.

Finally, perceptions of well-being have been identified as being one of the most important variables for adolescent mental health (Casas, 2011). The associations between young people's self-control and well-being are currently unknown. Self-control can be identified as a key developmental skill, part of the broader self-regulation system, which plays an important role in how young people manage emotions (e.g., frustration and anger), their impulse reactions to situations, and how they persist with their goals (Baumeister and Heatherton, 1996). The importance of self-control for specific facets of well-being, such as mental health (Li et al., 2020), depression (Özdemir et al., 2014), and happiness (Cheung et al., 2014) have been examined. For instance, Özdemir et al. (2014) found that participants (aged 16–24 years) with low self-control reported higher levels of depression and loneliness, compared to those with high self-control. More specifically, in adolescents (aged 12–18 years) Orúzar, Miranda, Oriol, and Montserrat (Orúzar et al., 2019) examined the relationship between self-control and well-being, using multiple measures of well-being (e.g., Personal well-being Index and Overall Life Satisfaction). They reported a positive significant relationship, whereby high self-control was associated with higher subjective well-being, specifically from the Students Life Satisfaction Scale (Huebner, 1991). However, this study's sample focused on adolescents in residential care, as such, the findings may not be representative of the entire adolescent population. It is therefore important to further examine the associations between self-control and well-being in young people, this could subsequently inform interventions focused around healthy behaviors. Additionally, young people are at a critical developmental stage, in which well-being and self-control are rapidly developing (Casey et al., 2011; Sawyer et al., 2018). Understanding these associations early, whilst well-being and self-control are evolving, may help to design appropriate interventions with long-term benefits.

### 1.3 Aims and hypotheses

The research to date suggests that self-control could be of particular importance for physical activity, physical fitness, motor competence, cardiometabolic health and well-being in young people; yet these important associations are poorly understood. Therefore, the current study aims to provide a more complete understanding of the associations of self-control with physical activity, physical fitness, motor competence, cardiometabolic health, and well-being, through a cross-sectional, exploratory design. It is hypothesized that young people with high self-control will have higher levels of physical activity, greater physical fitness, higher motor competence, decreased prevalence of cardiometabolic disease risk factors, and higher self-report well-being, when compared with young people with low self-control. The present study offers a novel contribution to the current literature by examining the importance of self-control for objective health measures (blood pressure, blood glucose and plasma insulin), different components of physical fitness (motor and musculoskeletal fitness), motor competence, and well being.

## 2 Method

### 2.1 Study design

**Figure 1:**
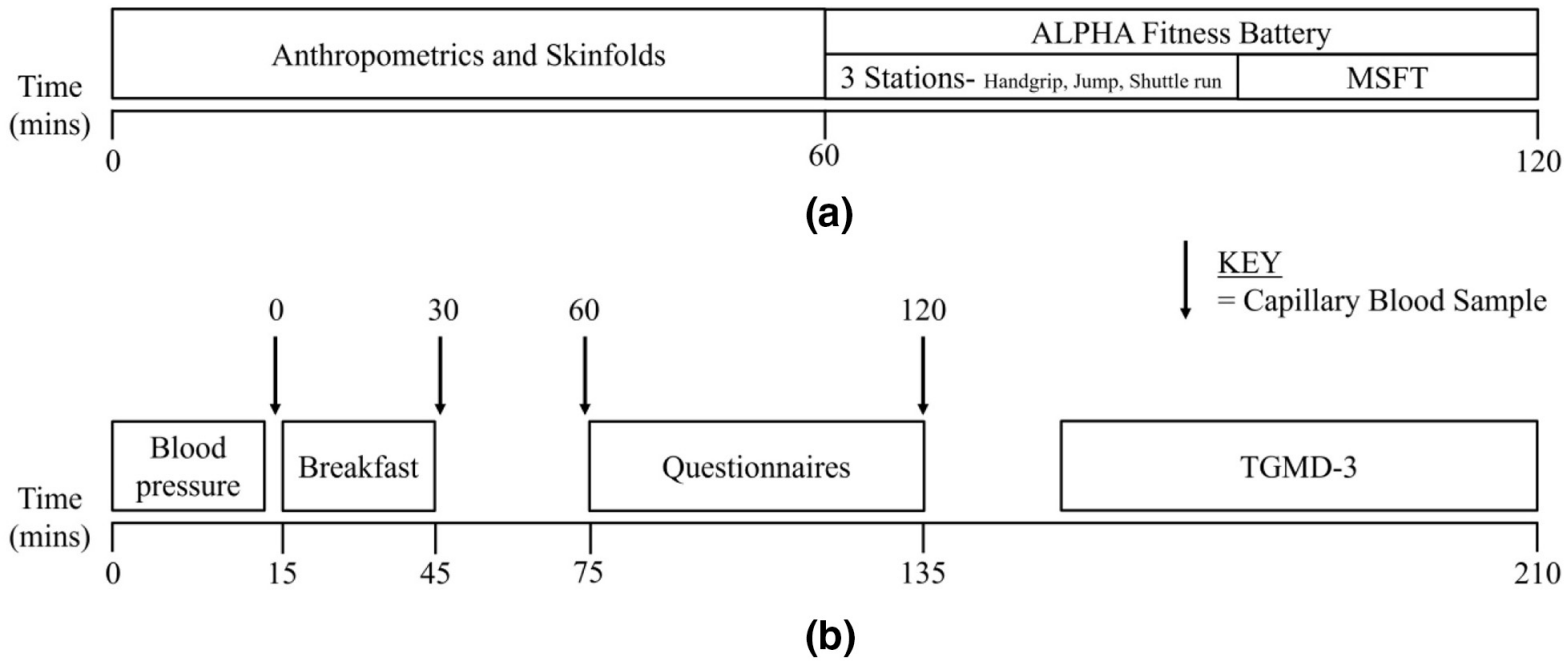
**(a)** Schematic of trial 1. **(b)** Schematic of trial 2.

Data collection took place between May 2022 and July 2023, within the school academic calendar. Participants took part in two trials, a minimum of 7 days apart. The first experimental trial (~2 h) consisted of anthropometric measurements and the ALPHA Fitness Battery (multi-stage fitness test, handgrip strength, standing broad jump, and 4 x 10 m shuttle run; see Figure 1a). Participants were provided with an accelerometer after the first experimental trial and instructed to wear for 7 days. Upon arrival to the main experimental trial, participant's fasted and resting blood pressure and a capillary blood sample were taken. Participants subsequently consumed a standardized breakfast (toast with margarine, cornflakes and milk), containing 1.5 g carbohydrate per kg body mass, as previously used (Cooper et al., 2018). well-being and self-control questionnaires were administered (see measures section), followed by postprandial capillary blood samples (30-, 60-, and 120-min post-breakfast). Participants also completed the motor competence tests (for details see measures section). An overview of the experimental protocol is shown in Figure 1b.

The study was approved by the institution's ethical advisory committee (Application number: 744; Date of Approval: 27th January 2022) and school-level consent was provided from the Headteacher. Parent/guardians provided written informed consent and completed a health screen questionnaire on behalf of the participant, ensuring they were in good health and had no medical conditions affecting their participation in the study. Participant assent was obtained after an explanation of the study.

### 2.2 Participants

The present study recruited a cross-sectional sample of 149 participants, from primary and secondary schools (9–13 years) in the East Midlands, England. Participants were recruited through convenience sampling, with school participation determined by willingness to participate. The schools represented a mix of urban and suburban catchment areas and had varied socioeconomic profiles. This regional and socioeconomic diversity should be considered when interpreting the findings generalizability beyond the East Midlands. During the first experimental trial, measurements for height (Leicester Height Measure, Seca, Hamburg, Germany), sitting height, and body mass (Seca 770 digital scale, Hamburg, Germany) were taken (see Table 1). To calculate maturity offset, the method by Moore, McKay, Macdonald and Nettlefold (Moore et al., 2015) was utilized.

**Table 1:** Participant descriptive characteristics (mean ± standard deviation), categorized by sex.

Variable	Overall (*n* = 149) Mean ±Standard deviation	Boys (*n* = 69) Mean ±Standard deviation	Girls (*n* = 80) Mean ±Standard deviation
Age (y)	12.1 ± 1.3	12.0 ± 1.0	12.2 ± 1.4
Height (cm)	155.4 ± 9.5	155.8 ± 9.4	155.0 ± 9.7
Sitting height (cm)	81.5 ± 5.3	80.5 ± 4.9	82.4 ± 5.5
Body mass (kg)	46.0 ± 10.3	45.9 ± 10.2	46.2 ± 10.5
BMI (kg·m^−2^)	18.9 ± 3.0	18.7 ± 2.3	19.0 ± 3.0
BMI centile^a^	56.4 ± 30.2	59.6 ± 30.4	53.7 ± 30.0
Maturity offset (y)	−0.3 ± 1.5	−1.1 ± 1.6	0.4 ± 1.0

^a^calculated based on national reference values (Cole et al., 2000)

### 2.3 Measures

A range of validated measures were used to assess self-control, physical fitness, cardiometabolic health, motor competence, adiposity, and well-being in young people. Self-control was measured using the Trait Self-Control Scale. Physical fitness was assessed using the multi-stage fitness test, standing broad jump, handgrip strength, and the 4 × 10 m shuttle run. Adiposity was evaluated through body mass index, triceps and subscapular skinfolds, and waist circumference. well-being was measured using the KIDSCREEN-27, and motor competence using the Test of Gross Motor Development−3rd Edition. Cardiometabolic health was assessed via blood pressure, blood glucose, plasma insulin, and HOMA-IR. Detailed descriptions of each measure are provided below.

#### 2.3.1 Physical activity

Physical activity was measured via accelerometry over a 7 d period, using ActiGraph GT3X accelerometers (Actigraph; Pensacola, FL, USA). Participants were fitted with an accelerometer on their right hip and were instructed to continuously wear the accelerometer for 7 d, except for water-based activities (e.g., swimming). The accelerometers were initialized at a sampling rate of 90 Hz.

Data were downloaded using Actilife Software (V6 13.4; ActiGraph, Pensacola, FL, USA) and saved in raw file format (.gt3x files), before being converted to.AGD files (15 s epochs). Analyses of physical activity were performed on 127 participants, as 22 were removed from analyses due to not meeting the wear time classification of four days (>10 h per day), as determined from an algorithm by Choi, Ward, Schnelle & Buchowski (Choi et al., 2012) classifying non-wear time. Assumed sleep periods were removed during data processing (23:00–06:00). Count cutoffs by (Romanzini et al. 2014) were used to classify activity and summarized as follows: Sedentary ( ≤ 180 counts^.^15 s^−1^), Light physical activity (181–756 counts^.^15 s^−1^), Moderate (757–1111 counts^.^15 s^−1^), Vigorous (>1112 counts^.^15 s^−1^). The sum of the time spent in moderate, and time spent in vigorous physical activity was also calculated (>757 counts^.^15 s^−1^).

#### 2.3.2 Self-control

The 36-item Self-Control Scale was used as a measure for participants trait self-control (Tangney et al., 2018). Participants rated the degree to which they agreed with the items on a five-point Likert scale, ranging from 1 (*not at all)* to 5 *(very much)*. To ensure suitability for the study population, some items were rephrased (e.g., “iron” self-discipline was rephrased to ‘strong' self-discipline). The questionnaire has been found to have good reliability (Cronbach's α = 0.89) and re-test reliability (*r* = 0.89; Tangney et al., 2018). The brief form of this scale (13 items) has previously been used successfully in similar research (Boat et al., 2022) and has been found to carry face validity in a young population (Duckworth and Seligman, 2006), with Cronbach' alpha (α) = 0.86, indicating good internal consistency. A total score for trait self-control was generated (ranging from 36 to 180). Higher scores reflected higher levels of self-control.

#### 2.3.3 Physical fitness

*The extended ALPHA fitness battery*. The Extended ALPHA Fitness Battery (Ruiz et al., 2011) was used to provide a holistic overview of physical fitness. The ALPHA fitness battery is widely adopted across Europe to assess physical fitness in children and adolescents, and includes measures associated with cardiometabolic risk. It has been found to be a valid, reliable, feasible, and safe assessment of health-related physical fitness (Kolimechkov, 2017; Ruiz et al., 2011). The ALPHA fitness battery consisted of the following measures, all performed in accordance with guidelines (Ruiz et al., 2011).

*Cardiorespiratory fitness*. To assess cardiorespiratory fitness, the Multi-Stage Fitness Test (MSFT), consisting of repeated shuttle runs between two lines, 20 m apart, was completed by participants. The test started at 8.0 km·h^−1^ and increased to 9.0 km·h^−1^ after the first level; with subsequent levels increasing by 0.5 km h^−1^. An audio signal dictated the running speed (Ramsbottom et al., 1988). The aim was for as many shuttle runs as possible to be completed before either; the participant could not follow the set pace of the audio sound, the participant did not cross the line prior to the audio sound (3 consecutive times), or the participant chose to stop voluntarily. Encouragement was provided throughout the test from investigators and the test itself was paced by an investigator familiar with the test. This test has successfully been administered in a similar population in previous research (Dring et al., 2019; Matsudo et al., 2015).

*Musculoskeletal fitness*. To assess musculoskeletal fitness, participants performed a test of handgrip strength. Following adjustment of the handgrip for each participant, they were instructed to hold the handgrip dynamometer in their dominant hand, with their arm fully flexed and slightly away from the body. Participants performed a maximal isometric effort for 3 s. Verbal encouragement was provided by researchers. Participants performed three handgrip trials and the criterion measure was the maximum force achieved. If the participants third trial was the highest, another attempt was performed. Handgrip strength has previously been used in similar research, in a similar study population (Ulrich, 2016). In line with the ALPHA fitness battery, absolute handgrip strength was used in the subsequent analysis.

For the second measurement of musculoskeletal fitness, participants performed a standing broad jump (Castro-Piñero et al., 2010). Participants stood with their feet behind a taped line and using a two foot take off, jumped as far as possible, then landed with two feet. A tape measure measured the distance between the take-off line and the point the heel, that was nearest to take-off line, landed on the ground. Participants completed two jumps and the best attempt was recorded.

*Motor fitness*. To assess motor fitness, the 4 x 10 m shuttle run test was performed (Council of Europe, 1988; Ruiz et al., 2011). Participants sprinted between two lines marked 10 m apart. Starting 0.5 m from the start line, when instructed by a researcher, the participant sprinted to the opposite marker, turning, and returning to the start line, passing through timing gates situated on the start line (Brower Timing Systems IRD-T173, Draper, UT, USA). Participants completed two trials, with the fastest completion time used as the criterion measure.

*Body composition*. The selected methods for assessing body composition were waist circumference and skinfold thickness (International Society for Advancement of Kinanthropometry; Stewart A, 2011). Waist circumference was measured at the narrowest point between the xiphoid process of the sternum and the iliac crest. Skinfold thickness measures were taken at two sites (triceps, subscapular) to assess the body composition of the participants. Two measures were recorded and the mean of the two values were used for analyses. A third measurement was conducted if there was >5% discrepancy between the two values, and the median value was used instead.

#### 2.3.4 Motor competence

To assess gross motor competence, participant's completed the Test of Gross Motor Development 3rd Edition (TGMD-3; 42). The test consisted of a series of exercises to assess locomotor (6 tasks) and ball (7 tasks) skills. The locomotor skills involved; run, gallop, hop, skip, jump, slide. The ball skills involved; two-hand strike of a stationary ball, forehand strike of a ball, one-hand stationary dribble, two-hand catch, kick a stationary ball, overhand throw, and underhand throw. Motor performance was observed through video recordings and subsequently evaluated based on predetermined qualitative performance criteria. A score of “1” was given for successful completion of the performance criteria, and “0” for an unsuccessful attempt. The performance criteria of three ball skills (kick, underarm throw, and overarm throw) were adapted to include an additional criteria point (hit the target), therefore the total score for the TGMD-3 was out of 106. The TGMD-3 has been shown to have strong construct validity and reliability in measuring gross motor skills in children (Magistro et al., 2020).

#### 2.3.5 Well-being

Participant's well-being was assessed using the Kidscreen-27 (Ravens-Sieberer et al., 2007), which assessed well-being across five dimensions: physical well-being (5 items), psychological well-being (7 items); social support and peers (4 items); school environment (4 items); parent relations and autonomy (7 items). For each item (e.g., “Thinking about last week have you felt fit and well”), participants responded on a 5-point Likert scale. The questionnaire has been found to have sound criterion validity and appropriate internal consistency, with Cronbach' alpha (α) = 0.73 (Ng et al., 2015; Stevanovic et al., 2013).

#### 2.3.6 Blood pressure

Prior to the blood pressure measurement, participants were seated for 5 min with their arm supported at heart level and hand relaxed. Two readings, separated by 2 min, were taken on the right arm using an automated Omron 1,300 blood pressure monitor (HBP-1300, Omron, Milton Keynes, UK). The criterion measure was the mean of the two measurements. A third measurement was taken if the discrepancy between the two systolic measurements was > 5 mmHg. The median value was then used as the criterion measure. The mean arterial blood pressure was calculated using diastolic blood pressure + [0.33^*^(systolic blood pressure – diastolic blood pressure); Smeltzer et al., 2011].

#### 2.3.7 Capillary blood sample

To assess blood biomarkers (blood glucose and plasma insulin), capillary blood samples were taken in the fasted and postprandial state. These biomarkers were selected as early metabolic risk emerges during childhood and adolescence, thus they can act as risk detection (Weiss et al., 2004). To increase capillary blood flow, participants hands were submerged in warm water upon arrival to the laboratory and prior to every subsequent sample. Following breakfast consumption, fasted blood samples were taken using a single-use lancet (Unistik, Extra, 21G gauge, 2.0 mm depth, Owen Mumford Ltd). Subsequent blood samples were taken at 30 min, 60 min, and 120 min. Capillary blood was collected in EDTA coated microvettes (Sarstdet CB300, Sarstedt, Leicester, UK) and one single (20 μl) sample in a pre-calibrated glass pipette (Hawklsey and Sons Ltd, Sussex, UK). The 20 uL sample was deproteinized immediately in 200 μl ice-cooled 2.5% perchloric acid and centrifuged at 1000 x g for 15 min. The microvettes were centrifuged at 1000 x g for 4 min at 4°C (Eppendorph 5415C, Hamburg, Germany). Plasma was extracted and pipetted into 250 μl plastic vials, and then frozen at −20°C immediately, to be later transferred to −80°C as soon as possible, for subsequent analysis.

Blood glucose concentrations were measured in duplicate using a commercially available assay (GOD-PAP method, GL 2610; Randox). Plasma insulin concentrations were measured using a commercially available ELISA (Mercodia Ltd). The intra-assay coefficients of variation (%) for plasma insulin were 9.64%. Blood glucose and plasma insulin incremental area under the curve (iAUC) were calculated using previously described methods (Wolever and Jenkins, 1986). Insulin resistance was calculated using homeostatic model assessment for insulin resistance [HOMA-IR; fasted glucose (mmol^.^L^−1^) x fasted insulin (μU^.^mL^−1^)/22.5; Matthews et al., 1985].

### 2.4 Statistical analysis

The open access software RStudio was used to perform all analyses (RStudio Team, 2020). All variables of interest were visually inspected for the assumption of normality prior to modeling. To reduce multiple testing, Univariate Pearson's correlations were performed to assess the relationships between self-control and the variables of interest (MVPA, multi-stage fitness test, handgrip strength, standing broad jump, 4 x 10 m shuttle run, TGMD-3, maturity offset, sum of skinfolds [tricep and subscapular], BMI centile, waist circumference, mean arterial pressure, glucose iAUC, insulin iAUC, HOMA-IR, and Kidscreen-27). Best-subsets regression modeling was subsequently performed to identify the regression model that explained the most variance in self-control (models with the combinations of between 1 and 5 independent variables) using the *Leaps* R Package (Lumley and Lumley, 2004). It was constrained to report the best model for up to 5 independent variables, to ensure that models had sufficient events-per-variable based on the available sample size (Heinze et al., 2018). Model performance for each candidate model was subsequently assessed using the *Performance* R package (Lüdecke et al., 2021). From the 5 candidate models that were returned, the overall “best” model was determined as the one that maximized model fit (via adjusted *r*^2^) and minimized model error. Following from this, simple linear regression models were fit to investigate the relationship between sub-components of the Kidscreen-27 score and self-control. These approaches limited the number of models estimated and helped to maintain analytic discipline, however it does not eliminate the possibility of Type 1 errors. All data are presented as mean ± standard deviation, with statistical significance accepted at *p* < 0.05.

## 3 Results

For descriptive purposes, mean values of all variables, for all participants (split by sex) are displayed in Table 2.

**Table 2:** Well-being, physical activity, physical fitness, motor competence, adiposity, cardiometabolic health, and self-control values (mean ± standard deviation).

Variable	Combined Mean ±Standard deviation	Girls Mean ±Standard deviation	Boys Mean ±Standard deviation
Well-being			
Kidscreen-27	103 ± 14	99 ± 14	108 ± 13
Physical activity			
MVPA (min·day^−1^)	99.39 ± 40.50	82.33 ± 31.82	120.35 ± 40.39
Physical fitness			
MSFT (m)	860 ± 360	760 ± 300	980 ± 380
Handgrip (kg)	22.3 ± 5.9	22.8 ± 6.4	21.6 ± 5.1
Broad jump (cm)	148 ± 23	144 ± 22	152 ± 24
4 x 10 m Shuttle (sec)	12.1 ± 1.0	12.3 ± 0.9	11.9 ± 1.0
Motor competence			
TGMD-3 total	80 ± 11	77 ± 11	84 ± 10
TGMD-3 locomotor	34 ± 6	34 ± 6	35 ± 4
TGMD-3 ball	46 ± 8	43 ± 7	48 ± 8
Adiposity			
Sum of skinfolds (mm)	24 ± 12	25 ± 10	24 ± 14
BMI (kg^.^m^−2^)	18.9 ± 3.0	19.0 ± 3.0	18.7 ± 2.9
BMI centile	56.4 ± 30.2	53.7 ± 30.0	59.6 ± 30.4
Waist circumference (cm)	65.3 ± 7.8	63.5 ± 7.3	67.3 ± 7.9
Cardiometabolic health			
Mean arterial pressure (mmHg)	82 ± 9	83 ± 8	81 ± 11
Glucose iAUC (mmol·L^−1^ x 120 min)	99.5 ± 74.8	103.5 ± 73.8	94.0 ± 76.6
Insulin iAUC (pmol·L^−1^ x 120 min)	3,346.9 ± 2,439.4	3,591.3 ± 2,579.7	3,041.4 ± 2,241.0
HOMA-IR (AU)	2.5 ± 1.8	2.6 ± 1.6	2.4 ± 2.0
Self-control			
Self-control scale	111 ± 18	108 ± 19	114 ± 17

MVPA, moderate-vigorous physical activity; MSFT, multi-stage-fitness test; TGMD-3, test of gross motor development−3rd edition; BMI, body mass index; HOMA-IR, homeostatic model assessment of insulin resistance; iAUC, incremental area under the curve.

### 3.1 Individual models

Pearson's correlation analyses assessing the associations between self-control and physical fitness, motor competence, cardiometabolic health, and well-being are summarized in Table 3. There was a positive association between self-control and well-being (Kidscreen-27), whereby higher self-control was associated with a higher score on Kidscreen-27 (*r* = 0.43, *p* < 0.001). There were also positive associations between self-control and cardiorespiratory (distance run on the MSFT, *r* = 0.21, *p* = 0.012) and musculoskeletal fitness (standing broad jump, *r* = 0.19, *p* = 0.020).

**Table 3:** Pearson's correlation output, with 95% confidence intervals, for all independent variables with self-control.

Variable	Correlation coefficient (95% CI)	*p*
Well-being		
Kidscreen-27	0.43 (0.29, 0.55)	< 0.001^***^
Physical activity		
MVPA	0.11 (0.07, 0.28)	0.23
Physical fitness		
MSFT	0.21 (0.05, 0.36)	0.012^*^
Handgrip	−0.06 (−0.22, 0.11)	0.495
Broad jump	0.19 (0.03, 0.34)	0.020^*^
4 x 10 m shuttle	−0.23 (−0.38, −0.07)	0.004^**^
Motor competence		
TGMD-3 total	0.04 (−0.15, 0.22)	0.680
TGMD-3 locomotor	0.02 (−0.16, 0.19)	0.863
TGMD-3 ball	0.10 (−0.07, 0.27)	0.257
Adiposity		
Maturity offset	−0.04 (−0.20, 0.12)	0.665
Sum of skinfolds (tri and sub)	−0.07 (−0.23, 0.09)	0.406
BMI centile	−0.15 (−0.30, 0.01)	0.072
Waist circumference	0.002 (−0.16, 0.16)	0.982
Cardiometabolic health		
Blood pressure (MAP)	−0.10 (−0.26, 0.06)	0.230
Glucose iAUC	0.05 (−0.14, 0.24)	0.623
Insulin iAUC	−0.09 (−0.28, 0.10)	0.333
HOMA-IR	−0.05 (−0.22, 0.13)	0.599

^***^p < 0.001;

^**^p < 0.01;

^*^p < 0.05

CI, Confidence Interval

No associations were found between self-control and handgrip strength (*p* = 0.495), motor competence (total score on TGMD-3, *p* = 0.680, TGMD-3 locomotor score, *p* = 0.863, and TGMD-3 ball skills, *p* = 0.257), physical activity (*p* = 0.23), adiposity [sum of skinfolds (triceps and subscapular), *p* = 0.406, BMI, *p* = 0.072, maturity offset, *p* = 0.665, and waist circumference, *p* = 0.982] or cardiometabolic health (blood pressure, *p* = 0.230, glucose IAUC, *p* = 0.623, insulin IAUC, *p* = 0.333, and HOMA-IR, *p* = 0.599).

Negative associations between self-control and motor fitness (4 x 10 m shuttle run) were found, whereby higher self-control was associated with faster performance time on the 4 x 10 m shuttle run test (*r* = −0.23, *p* = 0.004).

### 3.2 Best subsets regression

The results of the best subsets regression analyses (i.e., the models with the combinations of between 1 and 5 independent variables that best explained the variance in self-control) are displayed in Table 4. The overall model which explained the greatest variance in self-control (with the highest adjusted *r*^2^) included well-being (total scored on Kidscreen-27), motor fitness (time on 4 x 10 m shuttle run), and homeostatic model of insulin resistance (Model 3, *r*^2^ = 0.27, Table 4).

**Table 4:** Best subset regression model that explains the greatest variance in self-control.

Model	Variable	Parameter estimate	Standard error	*t*	*p*
**Model 1**	***F*** **(1,146)** = **33.6**, ***p***<**0.001, Adj** *r*^2^ = **0.18**				
	Intercept	52.9	10.07		
	Kidscreen-27	0.6	0.1	5.8	< 0.001^*^
**Model 2**	***F*** **(2,145)** = **23.1**, ***p***<**0.001, Adj** *r*^2^ = **0.23**				
	Intercept	106.6	19.23		
	Kidscreen-27	0.6	0.1	6.0	< 0.001^*^
	4 x 10 m Shuttle	−4.5	1.4	−3.2	< 0.001^*^
**Model 3**	***F*** **(3,124)** = **16.88**, ***p***<**0.001, Adj** *r*^2^ = **0.27**				
	Intercept	103.4	20.7		
	Kidscreen-27	0.7	0.1	6.3	< 0.001^*^
	4 x 10 m shuttle	−4.9	1.5	−3.4	< 0.001^*^
	HOMA-IR	−0.1	0.8	−0.1	0.94
**Model 4**	***F*** **(4,141)** = **11.52**, ***p***<**0.001, Adj** *r*^2^ = **0.22**				
	Intercept	112.3	19.6		
	Kidscreen-27	0.6	0.1	5.7	< 0.001^*^
	4 x 10 m shuttle	−4.9	1.5	−3.4	< 0.001^*^
	BMI centile	−0.1	0.1	−1.4	0.17
	Sum tri and sub	0.2	0.2	1.3	0.20
**Model 5**	***F*** **(5,100)** = **6.02**, ***p***<**0.001, Adj** *r*^2^ = **0.19**				
	Intercept	114.9	23.8		
	Kidscreen-27	0.6	0.1	4.4	< 0.001^*^
	4 x 10 m shuttle	−5.1	1.7	−3.0	< 0.001^*^
	BMI centile	−0.1	0.1	−1.2	0.25
	Sum tri and sub	0.3	0.3	1.0	0.32
	Insulin AUC	0.0	0.0	0.2	0.88

^*^p < 0.001

### 3.3 Kidscreen-27 sub-components

**Figure 2:**
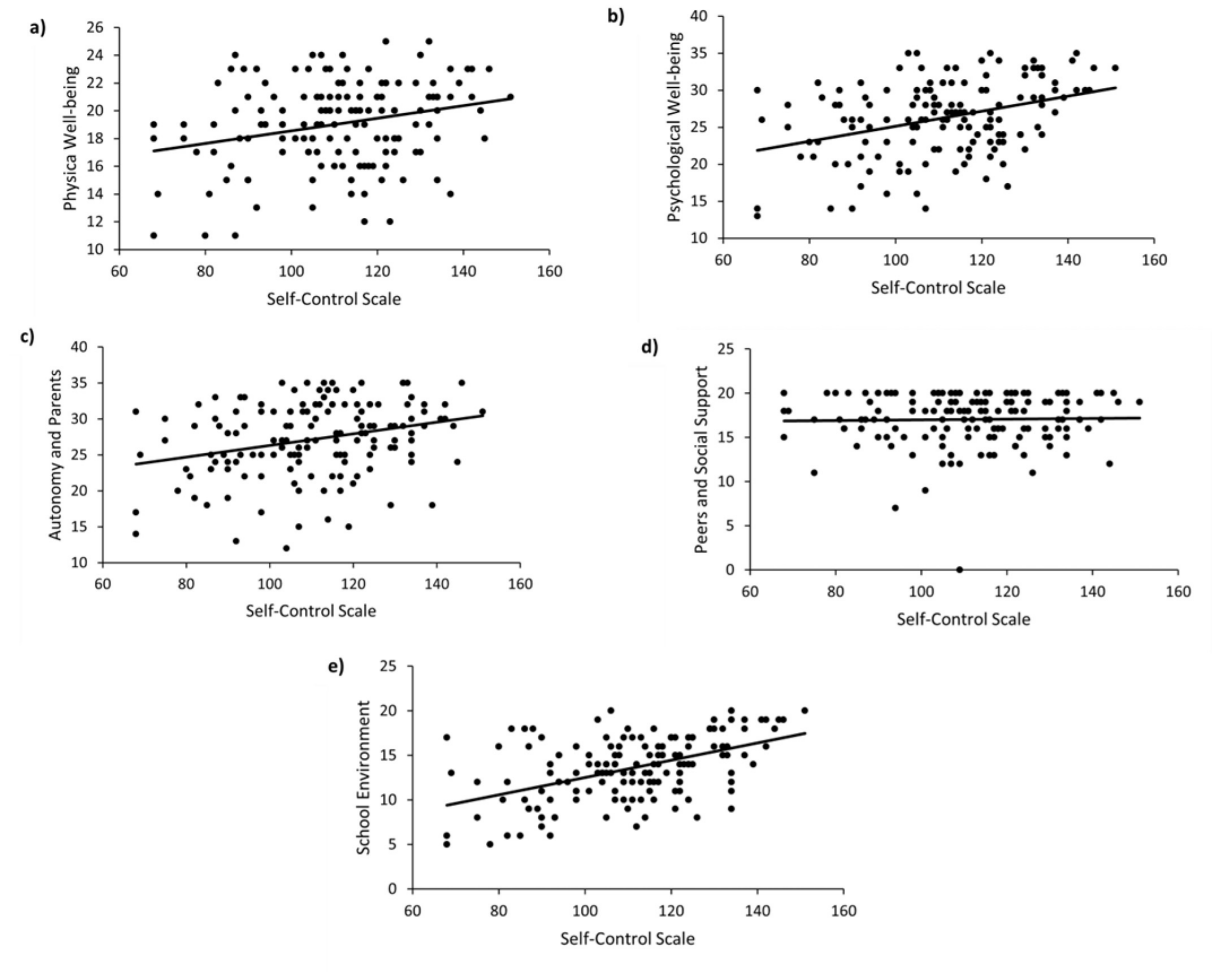
Cross-sectional associations between self-control (self-control scale) and individual components of well-being (Kidscreen-27). **(a)** Associations between self-control and physical well-being (*p* = 0.001). **(b)** Associations between self-control and psychological well-being (*p* < 0.001). **(c)** Associations between self-control and autonomy and parents (*p* < 0.001). **(d)** Associations between self-control and peers and social support (*p* = 0.790). **(e)** Associations between self-control and school environment (*p* < 0.001).

A summary of the outcomes of the linear regression models analyzing the relationship between self-control and sub-components of well-being are presented in Table 5. Significant positive associations were found between self-control and physical well-being (adjusted *r*^2^ = 0.064, *p* = 0.001, Figure 2a), psychological wellbeing (adjusted *r*^2^ = 0.131, *p* < 0.001, Figure 2b), autonomy and parents (adjusted *r*^2^ = 0.075, *p* < 0.001, Figure 2c), and school environment (adjusted *r*^2^ = 0.237, *p* < 0.001, Figure 2e) sub-components of the Kidscreen-27 questionnaire. No associations were found between self-control and the peers-and-social support sub-component (*p* = 0.790, Figure 2d).

**Table 5:** Simple linear regression models assessing the associations between self-control and individual components of well-being.

Variable	Intercept	Coefficient (95% CI)	Adjusted *r*^2^	*p*
Physical well-being	81.39	0.464 (0.625, 2.458)	0.064	0.001^*^
Psychological well-being	75.52	0.278 (0.794, 1.893)	0.131	< 0.001^*^
Autonomy and parents	83.48	0.278 (0.452, 1.549)	0.075	< 0.001^*^
Peers and social support	108.11	0.576 (−0.985, 1.292)	−0.006	0.790
School environment	77.01	0.364 (1.776, 3.214)	0.237	< 0.001^*^

^*^p < 0.001

CI, Confidence Interval

## 4 Discussion

The present study's main findings are that higher trait self-control was associated with higher motor fitness (measured by time taken to complete 4 x 10 m shuttle run), higher cardiorespiratory fitness (measured by distance ran on MSFT), higher musculoskeletal fitness (measured by distance on standing broad jump), and better well-being (higher score on Kidscreen-27) in young people. Specifically with regards to well-being, higher self-control was positively associated with several sub-components of well-being (physical well-being, psychological well-being, autonomy and quality of family relations, and school environment). When considering the best subsets regression, the model that accounted for the greatest variance in self-control consisted of well-being, motor fitness, and cardiometabolic health (measured by Kidscreen-27, 4 x 10 m shuttle run and HOMA-IR; respectively), with these effects occurring independently of one another. These findings highlight self-control as an important psychological variable, associated with favorable health outcomes in young people.

An important finding of the current study revealed that high self-control was associated with better well-being (higher score on Kidscreen-27). Whilst previous research has identified self-control's positive associations with mental health (Li et al., 2020) and happiness (Cheung et al., 2014), to our best knowledge, this study is the first to examine young people's self-control using a multi-faceted, holistic, approach to well-being. Given well-being was present in all models of the best subset regression, it highlights its important association with self-control. All sub-components in the Kidscreen-27, apart from peers and social support, displayed strong positive associations with self-control, demonstrating how self-control interacts with multiple domains of well-being, not just specific facets. High self-control was positively associated with the sub-component of physical health, indicating individuals with high self-control perceive their physical health to be good, compared to those with low self-control. This highlights the importance of self-control with health promoting behaviors and could contribute to young people adhering to an active healthy lifestyle. Previously, high self-control was reported to be associated with high emotional regulation (Tice and Bratslavsky, 2000), an ability which can contribute to greater psychological well-being; possibly explaining the association between psychological health and self-control in the present study. Furthermore, the study provides evidence that high self-control plays a significant role in a positive school environment experience. Given that self-control and the inhibitory control sub-set of executive function are often considered interchangeably, those with high self-control will also exhibit greater executive function; potentially leading to better academic performance (Peng and Kievit, 2020). Finally, the finding of high self-control being associated with better autonomy and parental relations is unsurprising, given that evidence suggests that individuals with high self-control exhibit better interpersonal relationships with others (Tangney et al., 2018). Conversely, the lack of association between self-control and the peers and social support domain contradicted previous research. Evidence demonstrated that low self-control is related to problematic relationships among friends (Boman et al., 2012). However, it must be considered that the previous study investigated a collegiate sample, who could have more established friendships, compared to the younger age group in the current study. Nonetheless, the present study provides important novel evidence of the associations between self-control and multiple facets of well-being in young people.

The research also identified a novel finding of the significant association between self-control and motor fitness. Individuals with high self-control performed a faster time on the 4 x 10 m shuttle run (indicative of greater motor fitness), compared to individuals with low self-control. This could be explained through self-control supporting young people to override their impulse to slow down, when the shuttle run task becomes uncomfortable, and to instead persist through the discomfort and perform optimally (Baumeister et al., 2007). These findings extend previous work examining the associations between self-control and cardiorespiratory fitness (Boat et al., 2022) and suggest that self-control is important for multiple domains of physical fitness in young people. The present study highlighted the association of high self-control and musculoskeletal fitness (as measured by the standing broad jump). This is in accordance with previous studies examining the association of self-control and musculoskeletal fitness (Kinnunen et al., 2012), yet offers a novel element, in that a young population has not previously been examined. The same underlying mechanism proposed for motor fitness may likewise account for the musculoskeletal fitness results. Interestingly, the present study also found that handgrip strength (musculoskeletal fitness) was not associated with self-control. The variance in these results could be explained by the standing broad jump being a skill that is more commonly carried out by this population, whereas handgrip strength is an isolated skill, that is not commonly executed by a young population. Nonetheless, the findings of the present study extend previous literature by demonstrating the associations between self-control and multiple components of physical fitness in young people.

Despite the aforementioned positive associations between self-control and physical fitness and well-being, the present study does not report any associations between self-control and cardiometabolic risk factors (blood pressure, glucose iAUC, insulin iAUC, and HOMA-IR). This could be attributed to the variability in young people's insulin iAUC, glucose iAUC, and HOMA-IR (Jeffery et al., 2012), which can fluctuate widely during puberty, due to developmental and hormonal factors, which could have potentially overshadowed and obscured the relationship with self-control. In addition, lifestyle factors, such as diet and stress levels (Brady and Matthews, 2006), could act as mediating factors that influenced these relationships as these are strong determinants of cardiometabolic health. Longitudinal and intervention studies examining self-control and cardiometabolic risk factors would help to provide a better understanding of the importance of self-control for cardiometabolic health in young people.

The present study's best subset regression analysis revealed the model containing well-being, motor fitness, and HOMA-IR best explained the variance in self-control. These findings indicate that self-control has the potential to influence young people's well-being and motor fitness, independently of each other, indicating the unique and multi-faceted contributions of self-control. Whilst there is no direct association between HOMA-IR and self-control, its presence in the model suggests that it might have an indirect effect on the relationship indirectly between self-control, well-being, and motor fitness. Thus, the current study contributes novel insights into the importance of self-control for a range of health behaviors and outcomes in young people.

Interestingly, the findings of the present study did not reveal any associations between self-control and physical activity in young people, however it did identify associations between well-being and physical fitness. This suggests that the mechanisms responsible for the association between self-control, physical fitness and well-being may rely on other pathways besides habitual activity levels. This could include psychological pathways such as high intrinsic motivation, more effective goal setting, and potentially enhanced cognitive functioning, for example better attention and working memory. Evidence has demonstrated that these psychological pathways themselves are associated with self-control (Converse et al., 2019; Inzlicht et al., 2014; Werner and Berkman, 2024; Wolff et al., 2016). Additionally, it is possible that the association between higher self-control and physical fitness reflects engagement in unmeasured muscle-strengthening activities. As hip-worn accelerometers cannot detect such behaviors (Ridgers and Fairclough, 2011), this may help explain the lack of association observed between self-control and device-measured physical activity. Furthermore, it could influence the relationship between self-control and physical fitness (Ortega et al., 2015), and well-being, independently of habitual activity levels that are captured by accelerometry. Whilst this finding does not support previous findings, it does highlight how self-control in young people may influence health behaviors and outcomes through different pathways.

The present study offers novel insight through the use of objective health measures, the inclusion of well-being, and the variety of components of physical fitness assessed alongside their associations with self-control. Nonetheless, it has its limitations. The present study is cross-sectional; therefore, this prevents the understanding of the directionality of the relationships, preventing causal interpretation. (Boat and Cooper 2019) previously determined that self-control and physical activity exhibit a bidirectional relationship. Therefore, the directionality of the associations mentioned here should be considered in subsequent intervention-based research. Furthermore, the present study utilized a self-report measure of self-control. Self-report measures may have limitations in young people that should be considered. However, it is currently the best measure of self-control available. Future research could look to develop an objective measure of self-control (e.g., Stroop task); however, such an approach requires development and validation prior to use in studies of this nature. The findings offered several practical implications. Interventions aimed at improving healthy behaviors may benefit from incorporating self-control components. Additionally, early identification of young people with low self-control could help support those at greater risk of developing unhealthy behavioral patterns and those that would benefit from early intervention.

### 4.1 Conclusion

In conclusion, the findings of the present study highlight the associations between self-control and a variety of health-related behaviors and outcomes, specifically including well-being, musculoskeletal fitness, cardiorespiratory fitness, and motor fitness in young people. Future work should thus explore interventions focused on training young people's self-control, and examine whether self-control can causally influence these health behaviors and outcomes, such as physical fitness and well-being, helping to clarify the directionality and underlying mechanisms of these relationships.

## Data Availability

The raw data supporting the conclusions of this article will be made available by the authors, without undue reservation.
